# Revisiting the Role of Lactic Acid Bacteria in Cacao Fermentation: From Traditional Paradigm to Functional Precision

**DOI:** 10.3390/foods15142415

**Published:** 2026-07-08

**Authors:** Tania María Guzmán-Armenteros, Armando Echeverría, Jenny Ruales, Luis Ramos-Guerrero

**Affiliations:** 1 Facultad de Ingeniería Mecánica y Ciencias de la Producción, Carrera de Ingeniería en Alimentos, Escuela Superior Politécnica del Litoral, Campus Gustavo Galindo, km 30.5 Vía Perimetral, Guayaquil 090902, Ecuador; tamaguzm@espol.edu.ec; 2 Universidad Católica Santiago de Guayaquil, Subsistema de Posgrados, Guayaquil 090508, Ecuador; neptali.echeverria@cu.ucsg.edu.ec; 3 Department of Food Science and Biotechnology, Escuela Politécnica Nacional, Quito P.O. Box 17-01-2759, Ecuador; jenny.ruales@epn.edu.ec; 4 Facultad de Ingeniería y Ciencias Aplicadas, Carrera de Ingeniería Agroindustrial, Grupo de Investigación en Alimentos y Agroindustria (GIAA), Universidad de Las Américas (UDLA), Quito 170124, Ecuador

**Keywords:** cocoa fermentation, lactic acid bacteria, microbial indispensability, functional redundancy, precision fermentation

## Abstract

The role of lactic acid bacteria (LAB) in cocoa bean fermentation has long been considered central within the traditional yeast–LAB–acetic acid bacteria (AAB) succession model. However, their functional necessity for successful fermentation remains debated. While yeasts and AAB are consistently associated with key transformations such as pulp degradation, ethanol formation, ethanol oxidation, heat generation, and internal bean modification, increasing evidence indicates that cocoa fermentation can proceed effectively under minimal, transient, or reduced LAB activity. This review re-examines the presumed indispensability of LAB using a function-based framework that distinguishes microbial contributions according to their relationship with fermentation completion and process optimization. By integrating evidence from controlled fermentations, microbial suppression studies, starter-culture research, and recent ecological and multi-omics analyses, this review suggests that LAB are better interpreted, under the conditions reported to date, as context-dependent modulators rather than universal core drivers of fermentation completion. LAB contribute to acid balance, citrate metabolism, mannitol production, microbial interactions, ecological stabilization, and quality modulation; however, available evidence indicates that the principal biochemical transformations defining fermentation completion can occur when yeast and AAB activities remain preserved. These conclusions should be interpreted with caution, as direct experimental evidence remains limited and is largely derived from controlled or small-scale fermentation systems. This function-oriented perspective shifts the focus from taxonomic recurrence to biochemical necessity and provides a rational basis for designing starter cultures centered on pathway coverage, metabolic performance, process consistency, and cocoa quality.

## 1. Introduction

Cocoa bean fermentation is a complex microbial-driven bioprocess traditionally described as a sequential succession involving yeasts, lactic acid bacteria (LAB), and acetic acid bacteria (AAB), whose combined metabolic activities contribute to pulp degradation, bean curing, flavor precursor formation, and the technological quality of cocoa and chocolate [1,2,3,4,5,6,7]. Within this classical framework, yeasts initiate fermentation through sugar conversion, ethanol production, and pulp degradation, while AAB oxidize ethanol into acetic acid, generating heat and promoting the biochemical transformations inside the cocoa bean that are required for proper fermentation [2,4,7,8]. In contrast, the precise contribution of LAB remains less clearly defined at the level of process indispensability, despite their recurrent detection and recognized metabolic activity during fermentation [4].

LAB have been associated with acidification dynamics, citrate consumption, mannitol production, microbial stabilization, and the formation of metabolites potentially linked to aroma development [2,4,8,9,10]. Commonly reported species include *Lactiplantibacillus plantarum* (formerly *Lactobacillus plantarum*), *Limosilactobacillus fermentum* (formerly *Lactobacillus fermentum*), *Levilactobacillus brevis* (formerly *Lactobacillus brevis*), and *Leuconostoc mesenteroides*, which are frequently detected during the intermediate stages of fermentation [11,12,13,14,15,16,17]. Based on their recurrence and metabolic versatility, LAB have often been incorporated into microbial succession models and starter-culture formulations, reinforcing the perception that they represent central contributors to fermentation performance [1,4,18].

However, recurrent presence does not necessarily demonstrate functional indispensability. Several laboratory- and pilot-scale studies have shown that cocoa fermentations conducted under restricted, reduced, or non-dominant LAB activity can still achieve key indicators of fermentation completion, including sugar utilization, ethanol oxidation, internal bean browning, fermentation index progression, and acceptable sensory characteristics in the resulting chocolate [19,20]. These findings suggest that LAB may influence specific aspects of fermentation performance without necessarily determining whether the process can reach its principal biochemical and technological endpoints [4,21].

This distinction is particularly relevant because microbial succession patterns involving LAB are not strictly conserved across regions, cocoa genotypes, fermentation practices, or environmental conditions. Studies conducted in Ghana, Brazil, Ivory Coast, Colombia, Indonesia, and other cocoa-producing regions have reported substantial variation in the timing, abundance, and species composition of LAB communities during fermentation [15,22,23,24,25,26,27,28]. In some systems, high or sustained LAB populations are observed, whereas in others, LAB appear transiently, at low abundance, or with delayed development, without necessarily preventing the production of adequately fermented cocoa beans [8,19,20]. Such variability suggests that microbial roles should be interpreted not only according to taxonomic recurrence, but also according to the biochemical functions required to sustain fermentation progression.

Despite these observations, the literature still lacks a clear framework for distinguishing between microbial groups that are required for fermentation completion and those that primarily modulate process performance, ecological stability, or sensory quality. This conceptual gap has allowed the traditional yeast–LAB–AAB succession model to remain influential as a descriptive ecological framework, while leaving unresolved whether all three microbial groups are equally necessary under diverse fermentation conditions. Addressing this distinction is essential for improving the interpretation of microbial ecology, refining starter-culture design, and advancing toward more functionally targeted cocoa fermentation strategies.

Accordingly, this review reassesses the role of LAB in cocoa bean fermentation by integrating evidence from controlled fermentations, microbial suppression studies, starter-culture research, and recent multi-omics analyses. The review distinguishes between microbial indispensability for fermentation completion and technological relevance for process stability, repeatability, microbiological safety, and sensory quality. By shifting the focus from taxonomic recurrence to biochemical function, this work proposes a function-oriented framework for interpreting microbial contributions and guiding the rational design of starter cultures for cocoa fermentation.

## 2. Materials and Methods

A structured literature review was conducted to identify studies addressing microbial ecology, metabolic functions, and experimental manipulation of microbial groups during cocoa bean fermentation. The search was performed in Scopus, Web of Science, PubMed, and Google Scholar, covering records available up to August 2025. The search strategy combined terms related to cocoa fermentation and microbial groups using Boolean operators. The core search string, adapted where necessary to match the syntax of each database, was: (“cocoa fermentation” OR “cacao fermentation”) AND (“lactic acid bacteria” OR “acetic acid bacteria” OR “yeast” OR “microbial succession” OR “metagenomics” OR “metabolomics” OR “starter culture” OR “citrate metabolism” OR “functional redundancy”).

Records retrieved from the different databases were combined, and duplicates were identified and removed using reference management software (Mendeley Desktop, v2.145.0, Elsevier, Amsterdam, The Netherlands), followed by manual verification based on title, authorship, and year of publication. The initial search yielded approximately 2000 records. Individual database contributions were not retained as separate counts during the original search; therefore, the reported total corresponds to the merged output across all databases prior to duplicate removal. After duplicate removal and screening based on title and abstract relevance, 526 studies were selected for full-text evaluation. Of these, 76 studies were included in the final qualitative synthesis. The screening and selection process is summarized in a PRISMA-style flow diagram (Supplementary Figure S1).

Studies were included if they met at least one of the following criteria: (i) investigated spontaneous or controlled cocoa bean fermentations, (ii) reported microbial composition, succession patterns, or fermentation-related metabolic outputs, or (iii) provided experimental, metabolomic, metagenomic, metatranscriptomic, or starter-culture evidence linking microbial groups to specific biochemical processes. Studies were excluded if they: (i) focused exclusively on isolated strains without a cocoa fermentation context, (ii) lacked measurable microbial, biochemical, technological, or sensory data, or (iii) addressed unrelated fermentation systems without transferable functional relevance to cocoa fermentation.

The methodological quality of included studies was assessed qualitatively according to the clarity of experimental design, reproducibility of methods, relevance of fermentation conditions, and consistency of findings with independently published evidence. This assessment was used to contextualize the strength of the evidence and identify potential sources of bias, rather than as a basis for formal quantitative weighting or exclusion.

To support literature retrieval, the AI-assisted academic search engine Consensus (Consensus NLP, Inc., Boston, MA, USA) was used as a complementary tool to refine search queries and identify additional potentially relevant peer-reviewed studies that may not have been captured by the primary database searches. Consensus was not used as a primary source for study selection or evidence interpretation. Articles identified through this tool were subjected to the same screening, eligibility, and quality-assessment criteria applied to records retrieved from the bibliographic databases.

Functional attribution was evaluated using a qualitative, criteria-based approach. Evidence was classified into three categories: (i) direct experimental evidence, including controlled fermentations, microbial suppression, exclusion, or inoculation studies; (ii) omics-based inference, including metagenomic, metatranscriptomic, metabolomic, or pathway-level analyses linking taxa to functional capacity or activity; and (iii) correlative observational evidence, including co-occurrence of microbial groups and metabolites in spontaneous fermentations. Of the 76 studies included, 52 were classified as correlative observational evidence, 14 as omics-based inference, and 10 as direct experimental evidence. This classification was used to distinguish between microbial recurrence, demonstrated functional capacity, and causal evidence of process dependence.

Because direct suppression studies provide the strongest evidence for evaluating microbial indispensability, particular attention was given to studies that experimentally reduced or manipulated LAB activity during cocoa fermentation. However, such studies remain scarce and are concentrated within a limited number of research groups [19,20]. Consequently, conclusions regarding LAB indispensability were interpreted cautiously and in combination with ecological, omics-based, starter-culture, and observational evidence. Throughout the review, microbial roles were therefore assessed by distinguishing between contributions required for fermentation completion and contributions involved in process optimization, ecological stability, and quality modulation.

## 3. Results

### 3.1. Historical Paradigm and Its Assumptions

The yeast–LAB–AAB succession model emerged from repeated culture-dependent observations reporting an early predominance of yeasts, followed by lactic acid bacteria (LAB) and subsequently acetic acid bacteria (AAB) across diverse cocoa-producing regions and fermentation systems [9,18,29,30]. Its influence derives from its capacity to provide a coherent ecological explanation for the major physicochemical changes occurring during fermentation. In this model, yeasts are primarily associated with sugar conversion, ethanol production, and pulp degradation; LAB with citrate metabolism, organic acid production, and microbial modulation; and AAB with ethanol oxidation, acetic acid formation, and heat generation [4,23,30,31,32]. These sequential activities broadly explain the transition from pulp degradation to acid accumulation, temperature increase, and bean curing.

However, the strength of this model lies mainly in its descriptive consistency rather than in direct evidence demonstrating the indispensability of each microbial group. Most early studies documented the recurrence, abundance, and temporal succession of microbial populations and related these patterns to metabolite formation. Comparatively fewer studies tested whether the absence of or reduction in a given microbial group prevents fermentation completion or compromises key bean-quality indicators. As a result, recurrent detection has often been interpreted as evidence of functional necessity, although the former does not establish the latter.

This distinction is particularly important in cocoa fermentation because microbial activity occurs predominantly in the pulp-bean mass, whereas the biochemical transformations defining bean curing take place inside the cotyledons. Microorganisms metabolize pulp substrates and generate ethanol, organic acids, water, and heat; these compounds then diffuse into the beans and trigger embryo death, membrane disruption, acidification, endogenous enzyme activation, polyphenol oxidation, and the formation of flavor and color precursors [4,23]. Therefore, the presence of a microbial group in the pulp environment does not, by itself, demonstrate that the group is structurally required for the internal biochemical transformation of the bean.

Within the classical framework, LAB have been assigned several ecological and technological functions, including pulp acidification, citrate utilization, mannitol production, pH-mediated microbial stabilization, and the generation of lactate that may serve as an additional substrate for AAB [1,10,13,33,34,35,36,37,38,39,40]. These activities are biologically plausible and, in several cases, have been demonstrated under cocoa fermentation conditions or in cocoa-pulp simulation systems [11,25,36,41,42]. Nevertheless, much of the available evidence establishes what LAB can contribute to the fermentation ecosystem rather than whether fermentation depends on their presence to reach completion.

Accordingly, the traditional succession model remains valuable as a general ecological description of cocoa fermentation, but it should not be interpreted as a hierarchy of microbial indispensability. Recurrence, metabolic activity, and technological relevance provide important evidence of ecological participation, yet they do not necessarily establish structural necessity. The historical paradigm is therefore better understood as a model of common microbial organization rather than definitive proof that yeasts, LAB, and AAB are equally required under all fermentation conditions.

### 3.2. Experimental Evidence Challenging LAB Indispensability

**Figure 1:**
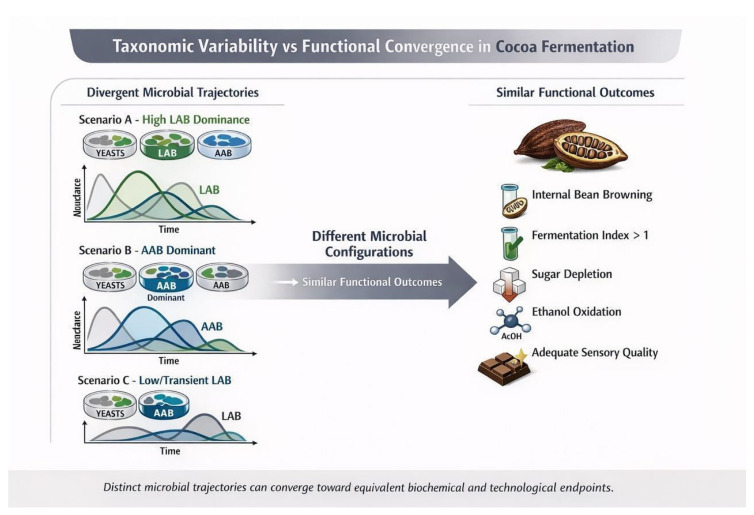
Taxonomic Variability and Functional Convergence in Cocoa Fermentation. Distinct microbial trajectories, including LAB-dominant, AAB-dominant, and low-LAB systems, converge toward similar core fermentation outcomes (bean browning, sugar depletion, acid formation, and quality-related outcomes). The scheme highlights that process success depends on functional execution rather than fixed microbial dominance.

The studies conducted by Ho et al. represent a critical advance in the evaluation of LAB functionality during cocoa fermentation because they moved the discussion from observational inference toward experimental validation [19,20]. Whereas the traditional yeast–LAB–AAB succession model largely inferred microbial importance from recurrence, abundance, and temporal succession, these studies directly manipulated microbial community composition through LAB suppression and simplified inoculated consortia. This experimental strategy allowed the consequences of reduced LAB participation to be evaluated under controlled fermentation conditions and provided one of the clearest opportunities to distinguish ecological association from functional dependence (Figure 1) [19,20].

A major strength of these investigations was their multidimensional assessment of fermentation performance. Rather than relying only on microbial counts or metabolite profiles, the authors evaluated microbial dynamics together with sugar consumption, organic acid accumulation, fermentation index, cotyledon browning, shell content, and sensory acceptance of the resulting chocolate [19,20]. This integrative design provided a stronger basis for interpreting microbial function than studies based exclusively on correlations between microbial abundance and biochemical changes.

Across the conditions examined, reduced LAB activity consistently altered specific metabolic outputs, particularly lactic acid production, but did not prevent the progression of the principal biochemical and technological transformations associated with successful fermentation [19,20]. Ethanol formation, acetic acid accumulation, temperature development, cotyledon browning, fermentation index progression, and acceptable sensory characteristics were maintained despite substantially reduced LAB populations. These findings challenge the assumption that recurrent abundance necessarily reflects functional indispensability and suggest that some fermentation-associated processes can be maintained through alternative microbial or physicochemical routes.

The implications of these findings extend beyond the specific case of LAB. They indicate that microbial dominance, metabolic contribution, and process dependence should be treated as distinct levels of evidence. A microbial group may be recurrent and metabolically active without being required for fermentation completion. This distinction is particularly relevant in cocoa fermentation, where many quality-related transformations result from the interaction between microbial metabolism in the pulp and endogenous biochemical reactions within the cotyledon.

Importantly, the studies also distinguish fermentation completion from fermentation optimization. LAB suppression did not prevent the attainment of key technological endpoints under the experimental conditions tested, but these studies were not designed to fully resolve the role of LAB in fine-flavor differentiation, aromatic complexity, ecological resilience, microbiological stability, or consistency under heterogeneous farm-scale conditions [19,20]. Therefore, current evidence supports the interpretation that LAB are not universally required for fermentation completion under the conditions reported to date, while still allowing for a significant role in quality modulation and process optimization.

Taken together, the work of Ho et al. provides the strongest direct experimental evidence currently available for reassessing the presumed indispensability of LAB in cocoa fermentation [19,20]. However, because this evidence derives from a limited number of controlled systems, its extrapolation to diverse cocoa genotypes, fermentation practices, and production scales should be approached cautiously. The broader value of these studies lies in redirecting the central question from whether LAB are consistently present to whether the functions attributed to LAB are uniquely dependent on this group or can be fulfilled by alternative microbial and physicochemical mechanisms within the fermentation ecosystem.

### 3.3. Ecological Variability and Functional Convergence

A consistent finding across the cocoa fermentation literature is the substantial variability of microbial communities among producing regions, fermentation systems, and processing conditions [8,16,17,23,42,43,44]. Differences in the abundance, timing, and succession of yeasts, lactic acid bacteria (LAB), and acetic acid bacteria (AAB) have been reported in fermentations from Ghana, Brazil, Ivory Coast, Colombia, Costa Rica, Ecuador, Indonesia, and other cocoa-producing regions [21,27,29,30,36]. Despite this taxonomic variability, successful fermentations frequently reach comparable technological endpoints, including pulp degradation, sugar depletion, organic acid accumulation, embryo death, cotyledon browning, and the formation of flavor precursors [21,30,33,34,45,46,47].

This convergence suggests that cocoa fermentation is not dependent on a single fixed microbial trajectory. Instead, different microbial configurations may support similar fermentation outcomes when the biochemical processes required for bean curing are preserved. Such observations are particularly important for interpreting the role of LAB, since their abundance and persistence vary considerably across fermentation systems without necessarily preventing the attainment of adequately fermented beans [8,19,20,27].

Microbial heterogeneity is also evident within individual fermentation masses. Fine-scale analyses have shown that oxygen gradients, temperature differences, pulp drainage, and turning practices generate spatially distinct microenvironments that can favor different microbial assemblages [27]. As a result, yeast, LAB, and AAB populations may vary locally while the batch as a whole continues to follow a coherent fermentation trajectory. This indicates that local taxonomic variation and overall process stability can coexist within the same fermentation system.

Differences in microbial community dynamics are also evident when comparing distinct fermentation systems at the process level. Spontaneous heap fermentations, box fermentations, and controlled or semi-controlled systems differ not only in temperature profiles, oxygen gradients, and turning frequency, but also in the timing, abundance, and species composition of the dominant microbial groups. Heap fermentations, widely used in West Africa, tend to support broader microbial diversity and more sustained LAB activity, whereas box fermentations, common in Latin America, are often associated with more structured successional patterns and earlier AAB dominance [24,28,29,36]. Controlled fermentations employing defined starter cultures or adjusted pulp content tend to produce more predictable community dynamics, often with reduced LAB persistence relative to spontaneous systems [1,19,20,48].

Despite these process-dependent differences in community structure, the principal biochemical endpoints associated with successful fermentation, including sugar depletion, ethanol oxidation, acid accumulation, cotyledon browning, and fermentation index progression, are consistently achieved across all three systems [8,21,30]. This pattern of functional convergence across fermentation systems reinforces the interpretation that fermentation completion depends more on the preservation of essential metabolic functions than on maintaining a specific microbial trajectory or succession pattern.

Taken together, these findings support the view that cocoa fermentation exhibits functional convergence across diverse ecological contexts. However, convergence should not be interpreted as complete equivalence among microbial communities. Differences in microbial composition can still influence pathway kinetics, metabolite balances, aromatic profiles, and sensory attributes. Thus, distinct microbial communities may support fermentation completion while differing in their efficiency, consistency, and impact on final cocoa quality.

### 3.4. Functional Redundancy and Metabolic Buffering

Multi-omics evidence suggests that cocoa fermentation is supported by a distributed metabolic architecture in which several central functions are shared among phylogenetically distinct microorganisms (Table 1). Although some biochemical transformations remain strongly associated with specific microbial groups, many pathways involved in fermentation progression are not confined to a single lineage. This organization helps explain why taxonomically distinct communities can maintain comparable biochemical trajectories when key metabolic functions remain active.

Metagenomic and metatranscriptomic studies have shown that pathways involved in carbohydrate degradation, pyruvate conversion, amino acid metabolism, and organic acid transformations can be encoded or expressed by yeasts, LAB, and AAB during cocoa fermentation [21,27,30,49,50]. These findings do not necessarily demonstrate complete functional replacement among taxa, but they provide evidence of functional overlap within the fermentation microbiota. Such overlap may buffer the process against fluctuations in the abundance of individual microbial groups.

This pattern is particularly relevant for central carbon metabolism. Glycolysis, pentose phosphate pathways, pyruvate metabolism, and multiple fermentative routes have been identified across diverse bacterial and yeast taxa associated with cocoa fermentation [21,30,50]. Although individual microorganisms differ in substrate preference, metabolic efficiency, and end-product profiles, the presence of parallel metabolic routes may help preserve carbon flux during community shifts.

At the same time, metabolic overlap is not uniform across all fermentation functions. Ethanol production is mainly associated with yeasts, particularly *Saccharomyces cerevisiae* and *Pichia* spp., whereas acetic acid production and thermogenesis are predominantly linked to AAB, including *Acetobacter* and *Gluconobacter* spp. [4,32,51,52,53,54]. These processes are closely connected to acid diffusion, embryo death, cotyledon browning, and the biochemical conditions required for flavor precursor development.

Several LAB-associated functions also show a higher degree of specialization. Lactic acid production is primarily linked to LAB, as yeasts generally do not contribute substantially to lactate accumulation under cocoa fermentation conditions [3,4,6,23,29,55]. Citrate degradation is also mainly attributed to LAB, and citrate lyase genes are only sporadically detected outside this group in cocoa-associated microbial communities [51,52,53]. Mannitol production likewise remains strongly associated with heterofermentative LAB, with limited evidence for major contributions from non-LAB taxa [21,51].

Taken together, the available evidence supports a model in which cocoa fermentation combines distributed metabolic capacity with group-specific functional specialization [41,56]. The reduction in a particular microbial population does not necessarily imply loss of fermentation functionality when overlapping pathways remain active. However, differences in community composition may still influence pathway efficiency, metabolite balance, reaction kinetics, and sensory outcomes. Functional overlap therefore provides a mechanism for process resilience, while specialized microbial activities continue to shape fermentation quality and consistency.

**Table 1:** Representative microbial taxa and evidence for functional overlap during cocoa fermentation.

Function/Pathway	Representative Taxa (Examples)	Evidence of Functional Overlap	Evidence Type	Sources
Ethanol production and pulp pectinolysis	*Saccharomyces cerevisiae*, *Hanseniaspora* spp., *Pichia fermentans*, *Kluyveromyces marxianus*, *Wickerhamomyces pijperi*	Distinct yeast consortia exhibit comparable sugar consumption kinetics, pulp degradation, and aroma precursor formation, indicating interchangeable fermentative capacity at the community level	Correlative/omics-based	[4,13,21,57,58]
Lactic-type fermentation (lactate and mannitol production)	*Limosilactobacillus fermentum*, *Lactiplantibacillus plantarum*, *Levilactobacillus brevis*, *Weissella ghanensis*	LAB species composition varies across regions and fermentations, yet heterolactic metabolism, citrate degradation, and mannitol pathways are conserved across multiple lineages	Correlative/omics-based	[54,56,59,60]
Acetate production from ethanol and lactate	*Acetobacter pasteurianus*, *Acetobacter tropicalis*, *Acetobacter aceti group*, *Gluconobacter* spp., *Komagataeibacter* spp.	Different AAB genera encode shared ethanol and lactate oxidation pathways, leading to convergent acetic acid production and thermogenesis	Correlative/omics-based	[56,57,59,60]
Pectin degradation and minor carbon source utilization	*Hanseniaspora* spp., *Pichia* spp., *Enterobacteriaceae, Bacillus* spp., *Pestalotiopsis* spp.	Multiple microbial groups possess pectinolytic and plant cell wall–degrading enzymes, suggesting redundancy in pulp breakdown capacity	Omics-based inference	[34,45,50,59]
General carbohydrate and amino acid catabolism	Diverse yeasts and bacteria within *Proteobacteria* and *Firmicutes*	Metagenomic analyses reveal similar functional gene repertoires across taxonomically distinct communities, supporting distributed metabolic potential	Omics-based inference	[56,57,59,61]

#### 3.4.1. Cross-Feeding Interactions and Distributed Network Architecture

Metabolic overlap in cocoa fermentation is reinforced by cross-feeding interactions that help stabilize carbon flow during shifts in community composition [7,41]. The most relevant example is the transfer of yeast-derived ethanol to AAB, which sustains acetic acid production and heat generation during the oxidative phase of fermentation [4,21,46,59,62,63]. Although lactate can serve as an additional substrate for some AAB populations, ethanol oxidation remains sufficient to support the main ethanol-to-acetic acid conversion pathway under typical fermentation conditions [21,30,45,50,59].

Other metabolites, including mannitol, organic acids, citrate-derived compounds, and intermediary carbon sources, may also circulate among microbial groups and contribute to metabolic flexibility [4,8,32,60]. These exchanges do not imply that all microorganisms are functionally equivalent, but they help explain how the fermentation ecosystem can maintain biochemical continuity despite changes in the abundance of individual populations.

Omics-based studies further support this interpretation by showing concurrent expression of genes involved in carbohydrate utilization and organic acid metabolism across multiple microbial groups during active fermentation stages [33,45,50]. Thus, cross-feeding should be interpreted not only as substrate exchange but also as part of a broader network architecture in which fermentation performance depends on the continuity of metabolic fluxes rather than on the dominance of a single microbial group.

#### 3.4.2. Minimal Functional Core

When cocoa fermentation is evaluated according to essential process outcomes rather than microbial composition, a relatively conserved functional core becomes apparent. Across diverse fermentation systems, successful fermentation is consistently associated with three interdependent biochemical events: conversion of pulp sugars into ethanol, oxidation of ethanol into acetic acid with concomitant heat generation, and diffusion of acid and heat into the cotyledons, leading to embryo death, internal browning, and precursor formation for flavor development [4,21,52].

Available evidence indicates that these core events are most closely associated with yeast and AAB activity. Yeasts drive ethanol formation through sugar metabolism, whereas AAB mediate ethanol oxidation, acetic acid accumulation, and thermogenesis [4,21,52]. These processes are strongly linked to fermentation index progression, cotyledon browning, acidification dynamics, and the biochemical conditions required for bean curing [21].

In contrast, LAB abundance appears less consistently associated with the attainment of these core endpoints. Acetic acid accumulation correlates more directly with ethanol availability and AAB activity than with LAB population size, while internal browning is principally determined by acid diffusion and temperature increase within the bean [21,53]. Similarly, although sugar depletion reflects the collective activity of the microbial community, available studies do not identify LAB abundance as a determining factor for reaching this endpoint [4,21].

Controlled fermentation experiments further support this interpretation, showing that biochemical completion can occur under very low or barely detectable LAB populations when yeast fermentation and AAB-driven oxidation remain active [19,20]. These observations suggest that the feasibility of cocoa fermentation depends primarily on preserving the biochemical sequence that drives bean curing, rather than on maintaining a fixed microbial succession pattern.

### 3.5. Network-Based Framework of Fermentation Stability

Recent systems-level and multi-omics studies have progressively shifted the interpretation of cocoa fermentation from a linear succession model toward a network-based view of microbial organization [21,27,30,50]. Within this framework, fermentation outcomes emerge from coordinated interactions among microbial populations rather than from the activity of isolated taxa.

This shift has important implications for how microbial roles are interpreted. Traditional fermentation studies have largely focused on identifying dominant taxa and describing succession patterns. However, accumulating evidence suggests that the biological relevance of a microbial group cannot be inferred solely from its abundance or persistence. Instead, the critical unit of analysis may be the metabolic function itself and the extent to which that function contributes to fermentation progression.

From this perspective, microbial communities can be viewed as collections of interacting functional modules rather than fixed taxonomic assemblages. Functions related to sugar conversion, ethanol oxidation, acid production, and precursor formation may be maintained despite substantial variation in community composition, provided that the underlying metabolic pathways remain active. This interpretation helps reconcile the apparent contradiction between highly variable microbial communities and the remarkable consistency of fermentation outcomes reported across production regions [21,27,30].

A network-based framework therefore shifts attention from the question of which microorganisms are present to which biochemical functions are preserved. Such a perspective provides a more mechanistic basis for evaluating microbial importance and may be particularly valuable for the development of next-generation starter cultures designed around functional performance rather than taxonomic composition alone.

### 3.6. Ecological Control and Microbial Stabilization Mediated by LAB and Phages

**Figure 2:**
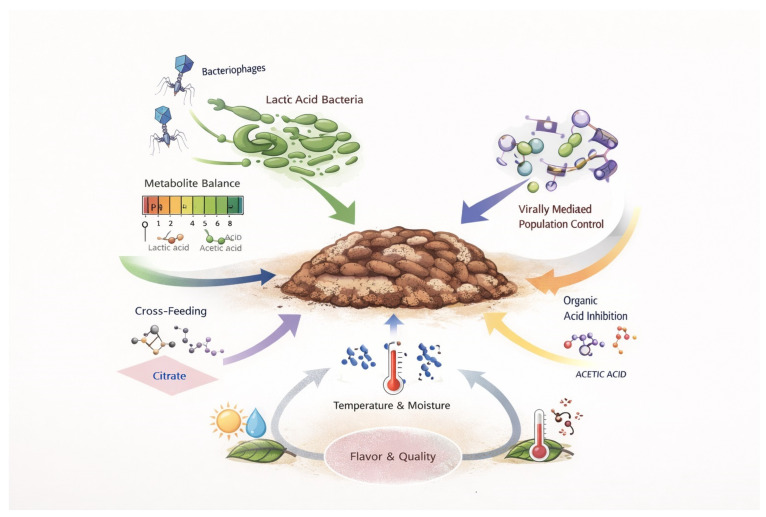
Multilayer ecological regulation in cocoa fermentation. Conceptual scheme illustrating regulatory processes that may shape microbial dynamics during cocoa fermentation. LAB contributes to acidification and antimicrobial activity, whereas bacteriophages may participate in bacterial population turnover. Solid arrows represent mechanisms supported by fermentation-related evidence; dashed arrows represent plausible but still insufficiently validated mechanisms. Together, these regulatory layers may contribute to community stabilization and preservation of fermentation functionality, although the direct role of phages in cocoa fermentation performance remains to be experimentally demonstrated.

A network-based view of cocoa fermentation also highlights the importance of ecological regulation in maintaining community stability (Figure 2). Processes such as environmental filtering, metabolic competition, antimicrobial activity, and viral predation may shape microbial succession and influence the preservation of functional activity under changing fermentation conditions.

Among the microbial groups involved, LAB contribute not only through metabolism but also through community modulation. Early acidification of the pulp environment promotes the decline of Enterobacteriaceae and other transient Gram-negative populations commonly associated with the initial stages of fermentation [4,37,64,65]. The resulting decrease in pH, together with organic acid accumulation, favors acid-tolerant microorganisms while limiting the proliferation of opportunistic or spoilage-associated taxa.

Several LAB strains isolated from cocoa fermentations have also shown antimicrobial activity under fermentation-relevant conditions [40]. Organic acids, hydrogen peroxide, and strain-dependent inhibitory compounds can suppress filamentous fungi and competing bacterial populations [40,66,67,68]. These effects may contribute to microbial stability and reduce the risk of undesirable metabolic activities, particularly in spontaneous fermentations exposed to environmental variability.

Ecological regulation, however, is not limited to LAB-mediated effects. Metagenomic studies have detected bacteriophages associated with dominant bacterial populations during cocoa fermentation [45]. Although these viral sequences usually represent a minor fraction of the detected community, they raise the possibility that phages may participate in bacterial population turnover and community restructuring [69,70,71,72]. At present, however, phage-mediated regulation in cocoa fermentation should be considered a plausible but unverified mechanism, because direct evidence linking phage activity to fermentation performance, pathway stability, or quality outcomes remains limited.

Taken together, current evidence indicates that microbial stabilization during cocoa fermentation emerges from multiple regulatory layers, including acidification, antimicrobial activity, environmental selection, competitive interactions, and possibly viral control. Within this framework, LAB contribute to ecological modulation and may enhance process consistency, but controlled suppression studies indicate that fermentation completion can still occur under reduced LAB abundance when the core biochemical processes associated with yeast and AAB activity remain preserved [19,20]. These observations reinforce the distinction between ecological relevance and functional indispensability within cocoa fermentation ecosystems.

### 3.7. A Function-Based Framework for Microbial Essentiality

The evidence reviewed supports interpreting microbial roles in cocoa fermentation according to their contribution to essential biochemical processes rather than their frequency of occurrence within succession patterns. From this perspective, microbial groups differ not only in their metabolic activities but also in the extent to which fermentation outcomes depend on those activities.

Microorganisms most closely associated with fermentation completion are those linked to the core biochemical sequence required for bean curing. Yeasts and acetic acid bacteria (AAB) are consistently involved in sugar conversion, ethanol formation, ethanol oxidation, acetic acid accumulation, heat generation, embryo death, cotyledon browning, and fermentation index progression [4,61,73,74,75]. These transformations represent the most reliable biochemical milestones of successful cocoa fermentation across production systems.

A second level includes functions that are distributed across multiple microbial groups and may therefore be partially buffered against taxonomic variation. Central carbohydrate metabolism, pyruvate conversion, and several organic acid transformations are encoded or expressed by diverse members of the fermentation microbiota, indicating functional overlap within the ecosystem [25,50,59,73]. These shared capacities may help preserve key metabolic fluxes when individual populations vary in abundance.

LAB occupy a more context-dependent position within this framework. The literature consistently supports their involvement in citrate metabolism, mannitol production, acid balance, microbial interactions, antimicrobial activity, and modulation of fermentation quality [52,53,76]. However, controlled suppression studies indicate that substantial reductions in LAB abundance do not necessarily prevent fermentation from reaching its principal biochemical and technological endpoints [19,20]. Therefore, current evidence suggests that LAB contribute importantly to process modulation, ecological stabilization, and quality development, while their role appears less directly tied to the feasibility of fermentation completion under the conditions reported to date.

This classification should not be interpreted as diminishing the technological relevance of LAB. Rather, it clarifies that ecological participation, metabolic contribution, and structural indispensability represent different levels of evidence. LAB may be highly valuable for improving process consistency, microbial safety, acid balance, and sensory performance, even if they are not universally required for the core biochemical sequence that defines fermentation completion.

Overall, a function-based framework provides a more precise basis for interpreting microbial importance in cocoa fermentation. It shifts the emphasis from whether a microorganism is recurrently detected to whether the biochemical functions associated with that microorganism are required, replaceable, or modulatory within the fermentation ecosystem.

## 4. Concluding Remarks

The evidence synthesized in this review supports a shift in the interpretation of cocoa fermentation from a taxonomically centered paradigm toward a function-oriented framework. Across diverse fermentation systems, successful fermentation appears to be more consistently associated with the preservation of key biochemical processes than with the recurrent dominance of specific microbial taxa. This perspective helps explain why fermentations with markedly different community compositions can nevertheless converge toward comparable biochemical and technological outcomes.

Within this framework, microbial importance should be evaluated according to the relationship between microbial activity and the biochemical transformations required for bean curing. Yeasts and AAB are most closely associated with the core sequence leading from sugar conversion to ethanol formation, ethanol oxidation, acetic acid accumulation, heat generation, embryo death, cotyledon browning, and fermentation index progression. LAB, in contrast, appear to contribute primarily to acid balance, citrate metabolism, mannitol production, microbial interactions, ecological regulation, and quality modulation. This interpretation does not reduce the technological relevance of LAB; rather, it suggests that their principal contribution may lie in improving fermentation stability, repeatability, microbial safety, and sensory performance rather than determining process feasibility itself.

The reviewed literature also indicates that cocoa fermentation operates as a resilient ecological system in which metabolic overlap, cross-feeding, environmental filtering, antimicrobial activity, and community-level regulation contribute to process stability. However, not all proposed regulatory mechanisms are equally supported. In particular, bacteriophage-mediated control remains a plausible but insufficiently validated mechanism in cocoa fermentation and should be addressed through targeted phage–host studies.

From an applied perspective, these findings support the development of starter-culture strategies based on functional coverage rather than taxonomic recurrence alone. Future formulations should prioritize the preservation of essential biochemical modules, including sugar conversion, ethanol oxidation, acid formation, thermogenesis, and precursor development, while also incorporating microorganisms that enhance consistency, safety, and sensory quality. LAB may therefore remain highly valuable in starter cultures, not because their inclusion is universally obligatory but because selected strains may improve process control and product differentiation.

Ultimately, the central contribution of this review is the distinction between microbial presence, functional contribution, and biochemical indispensability. The future of cocoa fermentation research may depend less on cataloguing which microorganisms are recurrently present and more on determining which functions are indispensable, which are partially replaceable, and which modulate quality. In this sense, functional necessity rather than taxonomic recurrence may provide the most informative framework for interpreting microbial contributions and designing next-generation cocoa fermentation strategies.

### Limitations and Future Research Needs

Several limitations should be considered when interpreting the conclusions of this review. First, the most direct evidence regarding LAB indispensability originates from a limited number of controlled suppression studies conducted predominantly by a single research group [19,20]. Although these studies provide valuable causal evidence, independent validation across different cocoa genotypes, fermentation practices, environmental conditions, geographic regions, and production scales remains limited. Therefore, the interpretation that LAB are not universally required for fermentation completion should be regarded as a well-supported working model rather than a universally established principle.

Second, the definition of fermentation success used in most studies relies primarily on biochemical and technological indicators such as sugar depletion, ethanol oxidation, acidification, internal browning, fermentation index progression, and the absence of visible spoilage. These parameters capture the essential transformations associated with fermentation completion, but they do not fully represent the sensory complexity, aromatic differentiation, and flavor refinement required for premium or fine-flavor cocoa. Consequently, current evidence is stronger for evaluating process feasibility than for determining the full contribution of LAB to sensory complexity and product differentiation.

Third, much of the evidence supporting functional convergence, metabolic overlap, and ecological resilience derives from observational, metagenomic, metatranscriptomic, and metabolomic studies. Although these approaches provide strong evidence of functional potential and pathway-level overlap, they do not always demonstrate causal substitutability among microbial groups. Future studies should therefore distinguish more explicitly between functional potential, functional expression, and experimentally validated functional compensation.

Finally, the ecological role of bacteriophages remains insufficiently characterized. Although viral sequences have been detected in cocoa fermentation ecosystems [45,69], their influence on microbial succession, community stability, metabolic fluxes, and fermentation performance has not yet been experimentally demonstrated. Integrated phage–host analyses are needed to determine whether viral processes merely reflect background microbial turnover or actively contribute to ecological regulation during fermentation.

Future research should prioritize multi-site suppression studies, controlled reconstruction of simplified microbial consortia, quantitative determination of functional thresholds, and integrated analyses combining metabolomics, metatranscriptomics, sensory evaluation, and phage–host interaction mapping. Such approaches will be essential for determining how far microbial functions can be redistributed among community members while preserving fermentation performance, process consistency, and chocolate quality.

## Data Availability

No new data were created or analyzed in this study. Data sharing is not applicable to this article.

## Supplementary Materials

The following supporting information can be downloaded at: https://www.mdpi.com/article/10.3390/foods15142415/s1, Figure S1: PRISMA-style flow diagram of study screening and selection. Records from the four databases were merged before deduplication. After duplicate removal and title/abstract screening, 526 studies were assessed in full text, and 76 were included in the final qualitative synthesis. The flow reflects a function-based evidence selection process distinguishing microbial recurrence, functional contribution, and evidence of process dependence.
