# Supplementary material for: Revisiting the Role of Lactic Acid Bacteria in Cacao Fermentation: From Traditional Paradigm to Functional Precision
Source: Foods. 2026 Jul 8;15(14):2415. doi: 10.3390/foods15142415 (PMC13407420; doi:10.3390/foods15142415)
Supplement: Supplementary file 1 [file foods-15-02415-s001.zip › foods-4373047-supplementary.pdf]

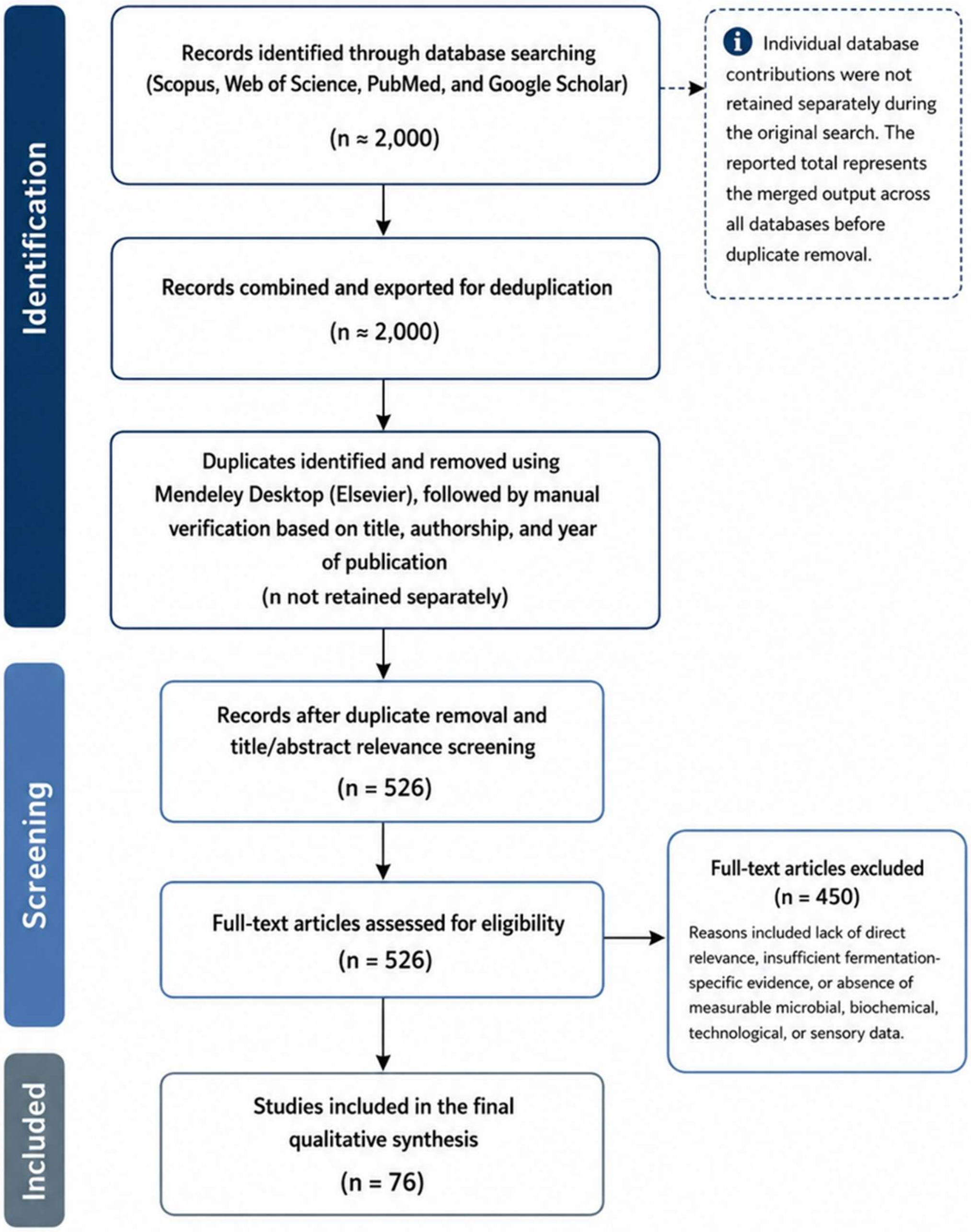

Figure S1. PRISMA-style flow diagram of study screening and selection. Records from the four databases were merged before deduplication. After duplicate removal and title/abstract screening, 526 studies were assessed in full text, and 76 were included in the final qualitative synthesis. The flow reflects a function-based evidence selection process distinguishing microbial recurrence, functional contribution, and evidence of process dependence.
